# Successful Pregnancy Outcome in Recurrent Ovarian Cancer in a 26 Year Old: A Case Report

**Published:** 2017-12

**Authors:** Savita Rani Singhal, Susheela Chaudhary, Kriti Agarwal, Anjali Gupta

**Affiliations:** Department of Obstetrics and Gynecology, PGIMS, Rohtak, India

**Keywords:** Ovarian, Cancer, Recurrent, Pregnancy, Chemotherapy

## Abstract

**Objective:** To report a case of Successful Pregnancy Outcome in Recurrent Ovarian Cancer in a 26 year Old.

**Case Report:** A 26 years old primigravida presented in antenatal clinic at 23 weeks of pregnancy with recurrence of ovarian cancer of mucinous type. Following refusal of surgical management, two courses of single dose carboplatin was administered. However, before third cycle of chemotherapy could be administered ,there was deranged liver functions tests, following which elective Cesarean section with staging laparotomy was planned at 34 weeks for breech presentation with oligohydramnios. A live healthy baby girl 2.3kg was delivered. Total abdominal hysterectomy with right salpingo-oopherectomy, infracolic omentectomy, appendectomy was done. The final diagnosis was recurrent mucinous ovarian adenocarcinoma. Postoperatively, she was given six cycles of chemotherapy (carboplatin and paclitaxel).

**Conclusion:** Chemotherapy and surgery, both are safe beyond first trimester and multidisciplinary treatment must be planned after taking into account the wishes of couple.

## Introduction

Incidence of gestational cancer is as low as 0.02% to 0.1% and it is even lower in developing countries because of the younger age of pregnant women (1-3). Cancer during pregnancy has become more frequent recently, because the number of women childbearing at an older age is increasing .Incidence of ovarian cancer is 1 in 15,000 to 1 in 32,000 pregnancies and it is the third most common malignancy in pregnancy, being preceded by cancers of cervix and breast (4, 5).

The clinical outcome of patients with epithelial ovarian cancer is not affected by pregnancy. Theauthors report a very rare case of recurrent ovarian cancer of mucinous type during pregnancy, managed successfully with combined modality of chemotherapy followed by surgery. This case is primarily being reported for rarity of recurrent ovarian cancer during pregnancy in developing countries.

## Case report

**Figure 1:**
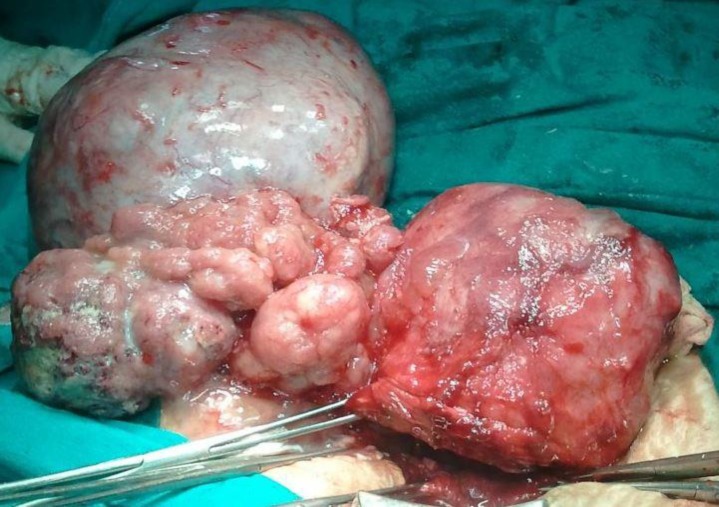
Intraoperative photograph showing right side tumor with rupture on the surface with tumor excrescences and uterus cut open after delivering the baby

A 26 years old primigravida unbooked case belonging to lower socioeconomic status, presented in antenatal clinic with 23 weeks of pregnancy with distension of abdomen. She had past history of left salpingo-oopherectomy done two years back for left ovarian mucinous adenocarcinoma. Postoperatively, she had received six cycles of combined chemotherapy with carboplatin and paclitaxel and was advised contraception. Subsequently she was lost to follow up until 23 weeks of period of gestation. Per abdomen examination revealed ascites and 24 weeks size uterus. with palpable fetal parts.CA-125 was 80 IU/ml. Ultrasound examination revealed intra-uterine pregnancy of23 weeks anda right ovarian mass of 12.8 x8.8 cm with solid and cystic areas containing free fluid with internal echoes. Diagnosis of recurrent ovarian cancer in pregnancy was made and she was advised surgical management which was declined by the patient. Hence two courses of chemotherapy with single dose carboplatin 450mg i.v. was started at three weekly intervals. However, third course of chemotherapy could not be administered due to derange liver functions tests. In spite of chemotherapy, ascites was progressive, leading to maternal respiratory distress and fetal growth restriction and oligohydroamnios ensued. Hence, elective Cesarean section with staging laparotomy was planned at 34 weeks for breech presentation with decreased liquor and gross ascites, after completion of steroid cover. Intraoperatively, 20litersof hemorrhagic ascetic fluid was drained, omentum was agglutinated over intestine and deposits were present over uterus. Right ovary was replaced by a tumor of 15x15cm with rupture and hemorrhage (Figure1).

A live healthy baby girl 2.3kg with APGAR 6/10 and 8/10 at 1 and 5minute respectively was delivered. The placenta appeared normal at the time of delivery. Total abdominal hysterectomy with right salpingo-oopherectomy, infracolic omentectomy, and appendectomy was done. Peritoneal washings were collected and multiple peritoneal biopsies were taken. Palpation of pelvic and para-aortic lymph nodes was negative. The patient had an unremarkable postoperative course. Histopathology revealed mucinous adenocarcinoma of ovary. She was given six cycles of chemotherapy (carboplatin and paclitaxel) commencing from postoperative day 14.At 6 month follow up, mother and baby are healthy.

## Discussion

Over 90% of the adnexal masses found in the first trimester disappear spontaneously. Teratomas, cystadenomas, endometriomas, ovarian cysts, and leiomyomas are the most frequent benign lesions (6). During pregnancy, malignant and borderline ovarian cancers account for 3% to 6% of cases and amongst them, the germ cell tumor is reportedly the most prevalent(50%) and epithelial ovarian cancer accounts for 20 % of all ovarian cancers (4-8).

The diagnostic method mainly depends on ultrasonographic findings because tumor markers during pregnancy are not helpful (6). Thus, surgery is mainly decided upon by the sonographic findings and clinical course (9). This was true in our case too, as CA125 was slightly raised which is normal during pregnancy and hence, patient was planned for definitive treatment based on sonographic findings of malignant ovarian mass, combined with obvious history of ovarian carcinoma and clinical evidence of gross ascites.

Delaying surgery increases the risk for bleeding, cystic rupture, and torsion whereas operating too early can increase the risk for luteal function loss and fetal loss (6). In our case, delaying surgery until fetal maturity lead to rupture of tumour with gross ascites. In first trimester, surgery is avoided except in cases of torsion due to risk of abortion. Moreover, chemotherapeutic agents are teratogenic in first trimester, risk being 83% but in second and third trimester risk of congenital malformed fetus is equal to general population (1.3%) (10). Owing to refusal of surgery by the couple, decision for administration of single agent chemotherapy with carboplatin was taken in our case. A study of 376 pregnant women reported the following after uterine exposure to chemotherapy: 5% cases of premature delivery, 7% cases of intrauterine growth restriction, 6% cases of fetal or neonatal death, and 4% cases of transient myelosuppression (11). In our case, iatrogenic preterm delivery was done due to fetal growth restriction, which was probably due to effect of cancer or chemotherapy. As in nonpregnant women, the regimen of choice for adjuvant chemotherapy is paclitaxel-carboplatin chemotherapy until fetal maturation. There is no convincing evidence that a synergistic increase in malformations occurs with the use of multiagent regimens as opposed to treatment with a single cytotoxic agent (12). Despite recurrence of ovarian tumour in pregnancy we resorted to the use of single agent carboplatin regimen until fetal maturity to minimize fetal toxicity and performed staging debulking laparotomy during Caesarean section followed by adjuvant chemotherapy postnatally.

This case posed great challenges. Firstly, it was a unique case of recurrence of epithelial ovarian cancer diagnosed during pregnancy. Since the patient was lost to follow up, it is difficult to determine if recurrence was preconceptional or gestational. Moreover, refusal of surgery in the second trimester by the couple was further compounded by inability to administer third cycle of chemotherapy on account of deranged liver function tests leading to tumor rupture. Fortunately, by this time, a stage had been reached where independent fetal survival was possible and decision for termination of pregnancy combined with debulking surgery was taken.

## Conclusion

Early finding of ascites by clinical or ultrasound examination combined with persistent large ovarian mass during pregnancy is strongly associated with advanced stage cancer. Moreover, pregnant women in advanced stage of ovarian cancer seem to have poor prognosis. Chemotherapy and surgery, both are safe beyond first trimester and multidisciplinary treatment must be planned after taking into account the wishes of couple.
